# Chronological Schedule of the Royal College of Surgeons

**Published:** 1844-01

**Authors:** 


### Chronological Schedule of the Fellows of the Royal College of Surgeons of England. (*First List.* 300.)

John Goldwyer Andrews, London.
Sir Benjamin C. Brodie, Bart. London.
Samuel Cooper, London.
Honoratus L. Thomas, London.
Robert Keate, London.
John Painter Vincent, London.
George James Guthrie, London.
Anthony White, London.
Thomas Copeland, London.
James Briggs, London.
William Lawrence, London.
Benjamin Travers, London.
Joseph Swan, London.
Edward Stanley, London.
Joseph Henry Green, London.
Thomas Callaway, London.
George G. Babington, London.
Robert Liston, London.
James M. Arnott, London.
John Flint South, London.
John Morgan, London.*

Robert R. Pennington, London.
Alexander Ogilvy, London.
Richard H. H. Steel, Berkhampstead.
Thomas Nixon, Pepperwick
James Borland, Teddington
Sir Simon Heward, Knt., Carlisle.
John Gunning, Paris.
William Hey, sen., Leeds.
Robert Bloxham, Isle of Wight.
Richard Cartwright, London.
Thomas Beckett, London.
Stephen Woolrich, Bridgnorth.
James Annesley, London.
George Frederick Albert, Cheltenham.
Henry Parkin, Woolwich.
Thomas Kidd, Corfu.
Joseph Constantine Carpue, London.
Sir James Pitcairn, Knt., Cork.
Henry Coates, Salisbury.
Sir Stephen L. Hammick, Bt., London.
George William Young, London.
Joseph Langstaff, London.
John Smith Soden, Bath.
Charles Seager, Clifton.
William H. Crowfoot, Beccles.
William Percival, Sen., Northampton.
George Norman, Bath.
Samuel Dyer, London.
John Bacot, London.
Richard Wood, Birmingham.
John Harris, Exeter.
John Badley, Dudley.
Bowyer Vaux, Birmingham.
Sir John Chapman, Knt., Windsor.
William Attree, Brighton.
George G. Campbell, London.
George Langstaff, London.
Samuel Ludlow, Exeter.
Jonathan Toogood, Bridgewater.
Sir Augustus West, Knt., Paris.
Morgan Thomas, Woolwich.
Thomas Davis, London.
William Andrews, Salisbury.
James Dawson, Liverpool.
John Ridout, London.
John Augustus Knipe, London.
Vero Clarke Kemball, London.
John Wright, Derby.
Hugh C. Standert, Taunton.
Charles Farrell, Dalyston.
John A. Ransome, Manchester.
Robert Bickersteth, Liverpool.
Charles Boutflower, Liverpool.
Kenrick Watson, Stourport.
John Bishop Estlin, Bristol.
George Champney, York.
James Ainsworth, Manchester.
Andrew Brown, Paris.
Richard B. Godwin, Derby.
Harry Blaker, Brighton.
William Tuckwell, Oxford.
William B. Lynn, London.
John Grenfell Moyle, Cheltenham.
William Goodlad, Manchester.
James P. Sheppard, Worcester.
John North, London.
Robert Thorpe, Manchester.
Charles Wingfield, Oxford.
John Lawrence, Brighton.
Thomas Martin, Reigate.
Richard W. Brown, Bath.
Richard Blagden, London.
Charles Mayo, Winchester.
Gideon A. Mantell, Clapham.
Samuel Barnes, Exeter.
William Cother, Gloucester.
John Haddy James, Exeter.
John Nedham, Leicester.
Matthew Pierpoint, Worcester.
William Rae, Chatham.
Joseph Hodgson, Birmingham.
Samuel Smith, Leeds.
Henry Terry, Northampton.
Alfred Jukes, Birmingham.
John Clarke, Dumfries.
John Cooper, Liverpool.
William L. Thomas, Hatfield.
Charles H. Phillips, London.
John Baird, Newcastle-upon-Tyne.
Thomas Joseph Pettigrew, London.
William Jackson, Sheffield.
Robert Tayler, Brighton.
John Green Crosse, Norwich.
William J. Wilson, Manchester.
William Cleoburey, Oxford.
William Hunter, *Guards.*
John Okes, Cambridge.
James Wardrop, London.
Montague Gosset, London.
Thos. M. Greenhow, Newcastle-on-Tyne.
Sir John Fife, Knt., Newcastle-on-Tyne.
James Ranald Martin, London.

* The first twenty-one are Life Members of the Council.

William Wright, Nottingham.
William Kingdon, London.
William C. Watt, Malta.
Robert Harrison, Dublin.
James William Braine, London.
George B. Knowles, Birmingham.
Robert Armstrong, Plymouth.
Thomas Turner, Manchester.
John Boutflower, Manchester.
Henry Giles Lyford, Winchester.
William J. Wickham, Winchester.
John W. Wilton, Gloucester.
Joseph P. Garlick, Leeds.
John Mawdsley, London.
Edward Tegart, London.
Sir John Webb, Knt., Woolwich.
Sir Jacob Adolphus, Knt., Cheltenham.
John Y. Arrowsmith, Shrewsbury.
Eusebius A. Lloyd, London.
Daniel H. Walne, London.
Henry Edward Burd, Shrewsbury.
Thomas Arthur Stone, London.
William Hey, jun., Leeds.
Richard Hughes, Stafford.
John Masfen, Stafford.
William MacKenzie, Glasgow.
Andrew White, London.
George Macilwain, London.
Thomas Paget, Leicester.
John Hopps, York.
George Buckley Bolton, London.
Robert Craven, Hull.
Robert Ceeley, Aylesbury.
Herbert Mayo, Boppart.
Philip C. De la Garde, Exeter.
Francis P. B. Samwell, London.
George Sampson, London.
Robert Wade, London.
John Prince Halton, Liverpool.
Richard Welbank, London.
John Scott, London.
Edward Cutler, London.
William MacKenzie, Edinburgh.
Isaac Hurst, Bedford.
Charles Aston Key, London.
John W. Johnson, Derby.
Douglas Fox, Derby.
James Syme, Edinburgh.
Richard T. Gore, Bath.
Cæsar H. Hawkins, London.
Richard D. Grainger, Norwood.
Frederick C. Skey, London.
John Harris, Bedford.
F. J. R. Hale Thomson, London.
James Luke, London.
John M. Banner, Liverpool.
Thomas P. Teale, Leeds.
William Eccles, London.
Benjamin H. Norgate, Norwich.
William F. Morgan, Bristol.
Frederick Huntington, Hull.
Price B. Hallowes, Canterbury.
David B. Major, Canterbury.
John C. Taunton, London.
Richard A. Stafford, London.
Bransby B. Cooper, London.
William Sands Cox, Birmingham.
Thomas Wormald, London.
George Pilcher, London.
John Bishop, London.
John George Perry, London.
George Simpson, London.
Gilbert W. Mackmurdo, London.
Francis Kiernan, London.
Henry Heath, Newcastle-on-Tyne.
Henry Russell, York.
John Harrison, Bristol.
Thomas Green, Bristol.
Richard Hey, York.
John Malyn, London.
George Gulliver, *Horse Guards Blue.*
Edward W. Tuson, London.
Henry Clarke, Bristol.
Richard Owen, London.
John Willimot.
William Coulson, London.
Joseph Jordan, Manchester.
Wilson Overend, Sheffield.
John Dalrymple, London.
Samuel Gregory, Sheffield.
Richard Middlemore, Birmingham.
Richard Partridge, London.
John Hilton, London.
William H. Bainbrigge, Liverpool.
Richard Quain, London.
George Mills White, Nottingham.
Thomas C. Buchanan, Gloucester.
Henry T. Chapman, London.
Edward Cock, London.
Henry Denne, Canterbury.
S. W. Langston Parker, Birmingham.
Samuel Solly, London.
Thomas Tatum, London.
Alexander Shaw, London.
John Adams, London.
Alfred Hamilton, London.
George T. Morgan, London.
Henry D. Carden, Worcester.
Samuel A. Lane, London.
John Avery, London.
Frederick Dover, London.
John Hobbs, London.
James Farish, London.
John Dickin, Shrewsbury.
William Pennington, London.
Henry C. Attenburrow, Nottingham.
D. W. Crompton, Birmingham.
Andrew M. M'Whinnie, London.
Edward Wallis, Hull.
Alfred Joshua Wood, Gloucester.
Benjamin Phillips, London.
Henry Cooper, Hull.
George R. Tatum, Salisbury.
Charles Beevor, London.
Henry Jackson, Sheffield.
George Busk, *Dreadnought.*
T. Wilkinson King, London.
John Godwin Johnson, Norwich.
Henry Thomas, Sheffield.
Henry Stubbs, Liverpool.
Charles Lestourgeon, Cambridge.
William Gill, Liverpool.
Rutherford Alcock, London.
Benjamin Travers, jun., London.

Nathaniel Smith, Bristol.
W. J. Erasmus Wilson, London.
Thomas Nunneley, Leeds.
Robert Hughes, Stafford.
William H. Fletcher, Gloucester.
John Harrison, London.
James Long, Liverpool.
Thomas B. Curling, London.
Frederick Le Gros Clark, London.
Carsten Holthouse, London.
John Farrar Crookes, London.
Archibald Dalrymple, Norwich.
Edward F. Lonsdale, London.
Henry Charles Johnson, London.
Henry James Johnson, London.
Cornelius Harrison Browne, Canterbury.
Charles H. Rogers Harrison, London.
Philip Bennett Lucas, London.
Dennis Embleton, Newcastle-on-Tyne.
Henry Hancock, London.
John Gay, London.
John Newton Tomkins, London.
Samuel Holmden Amphlett, Birmingham.
Charles Lewes Parker, Oxford.
Alexander Ure, London.
George Lewis Cooper, London.
William Morrison, Newcastle-on-Tyne.
George Newport, London.
George Viner Ellis, London.
James Duncan, Edinburgh.
Thomas Morton, London.
Edward John Chance, London.
Campbell Grieg De Morgan, London.
John Chippendale, London.
James Dixon, London.
James Paget, London.
Josiah Hammond, Cambridge.
Prescott Gardner Hewett, London.
Francis Hird, London.
Richard William Tamplin, London.
Charles Hawkins, London.
William Trew, London.
Henry Smith, London.

